# A new lefteye flounder of the genus *Crossorhombus* (Teleostei, Bothidae) from Penghu Islands, western Taiwan

**DOI:** 10.3897/zookeys.1273.167628

**Published:** 2026-03-13

**Authors:** Kunio Amaoka, Yo Su, Hsuan-Ching Ho

**Affiliations:** 1 Hokkaido University, Hakodate, Hokkaido, Japan Department and Graduate Institution of Aquaculture, National Kaohsiung University of Science and Technology Kaohsiung Taiwan; 2 International Doctoral Program of Marine Science and Technology, National Sun Yat-sen University, Kaohsiung, Taiwan International Doctoral Program of Marine Science and Technology, National Sun Yat-sen University Kaohsiung Taiwan; 3 Department and Graduate Institution of Aquaculture, National Kaohsiung University of Science and Technology, Kaohsiung, Taiwan National Museum of Marine Biology and Aquarium Pingtung Taiwan; 4 Taiwan Ocean Research Institute, National Institutes of Applied Research, Kaohsiung, Taiwan Hokkaido University Hakodate Japan; 5 National Museum of Marine Biology and Aquarium, Pingtung, Taiwan Taiwan Ocean Research Institute, National Institutes of Applied Research Kaohsiung Taiwan; 6 Australia Museum, Sydney, Australia Australia Museum Sydney Australia

**Keywords:** Biodiversity, ichthyology, identification key, sexual dimorphism, taxonomy, western Pacific

## Abstract

A new lefteye flounder, *Crossorhombus pescadores***sp. nov**., is described based on five specimens collected from Penghu Islands. This new species differs from the other four congeners by having a pair of small black spots on the caudal fin, a horizontal triangular bluish-black marking on the blind side of body in male, the front wings of the marking reaching the base of dorsal and anal fins, and a combination of morphometric characters, including a larger head, deeper body, smaller eyes, fewer dorsal- and anal-fin rays and lateral-line scales, and other characters. A key to all five nominal species of *Crossorhombus* is provided.

## Introduction

The flatfish genus *Crossorhombus* Regan, 1920 belongs to the family Bothidae and is characterized by having a generally short, high, and oval body covered by scales with a single row of long, comb-like spines along their posterior margin, a small mouth, eyes divided by a wide depression that increases in width with growth and wider in males than females, a bluish-black marking on the blind side of males, and a caudal skeleton provided with many grooves in the four all plates, or branches at the inner two plates (Norman 1934; Amaoka 1969, 2016). This genus is a small group with only four valid species occurring in the Indo-West Pacific Ocean (Hensley and Randall 1993; Hensley and Amaoka 2001; Shen and Wu 2011; Shao 2025), including *C. azureus* (Alcock, 1889), *C. howensis* Hensley & Randall, 1993, *C. kobensis* (Jordan & Starks, 1906), and *C. valderostratus* (Alcock 1890).

Recently, Amaoka and Ho (2019) and Amaoka (2019) reported all four species in Taiwan. Nevertheless, when investigating the fish species in the bycatch of the Silver-stripe round herring fishery in Chi-Kan of Penghu Islands, five bothid specimens were collected that agree with the diagnostic characters of *Crossorhombus* but unusually having a pair of black spots on the caudal fin and a triangular bluish-black marking with anterior wings on the blind side of body in males. The characters make these specimens outstanding from all four previously known species of *Crossorhombus*.

The aforementioned specimens are described here as a new species and compared with the other four known congeners. In addition, a key was presented to facilitate the identification of all five species, including the new species.

## Materials and methods

The types collected from the waters of Taiwan examined in this study are deposited at the Pisces collection of Museum of Marine Biology and Aquarium (**NMMB-P**) and Hokkaido University Museum (**HUMZ**), and additional comparative specimens deposited at NMMB-P, HUMZ and the Smithsonian Institution National Museum of Natural History, Department of Vertebrate Zoology, Division of Fishes (**USNM**) were used.

Counts and proportional measurements follow those of Hubbs and Lagler (1958) and Amaoka et al. (1993). Standard length (**SL**) and head length (**HL**) are used throughout. All measurements were made using calipers. The dorsal-, anal- and caudal-fin rays, and vertebrae were counted from digital radiograph machines at NMMB-P and HUMZ. The features of caudal rays and skeleton were observed from the radiographs.

## Taxonomy

### Crossorhombus pescadores sp. nov.

Taxon classificationAnimaliaPleuronectiformesBothidae

E1A9F422-4A34-5DF7-A96B-CD063C35A27B

https://zoobank.org/EA48AC13-1E5C-4CB7-92A9-48BFA150BED0

**Figure 1.**
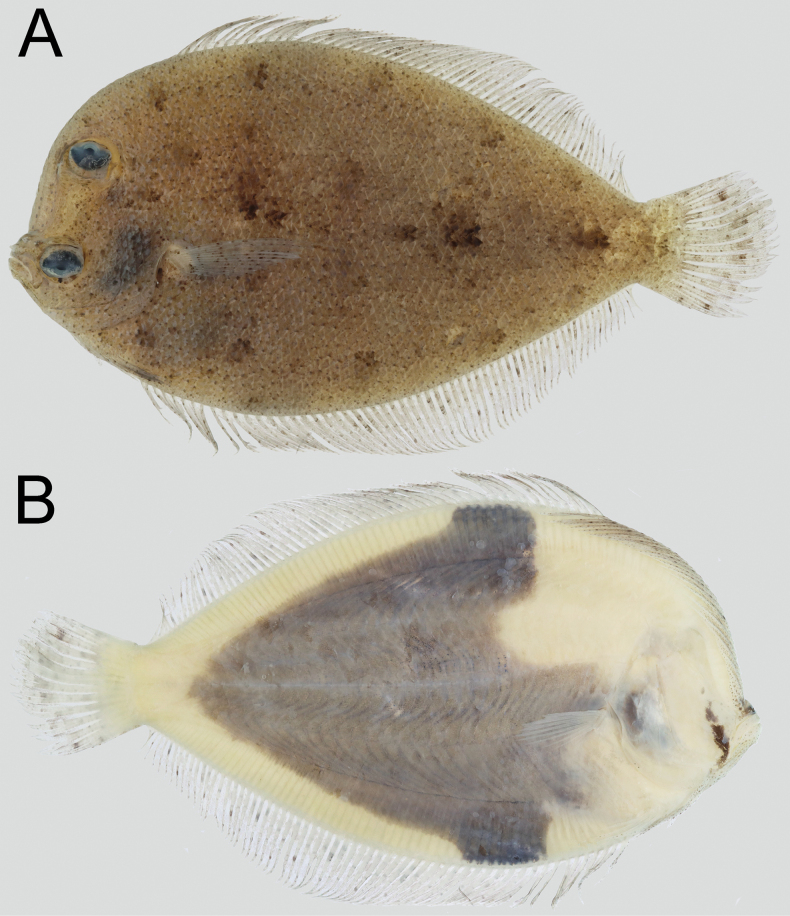
*Crossorhombus pescadores* sp. nov., NMMB-P 42260, holotype, male, 99.8 mm SL, preserved. **A**. Ocular side; **B**. Blind side.

**Figure 2.**
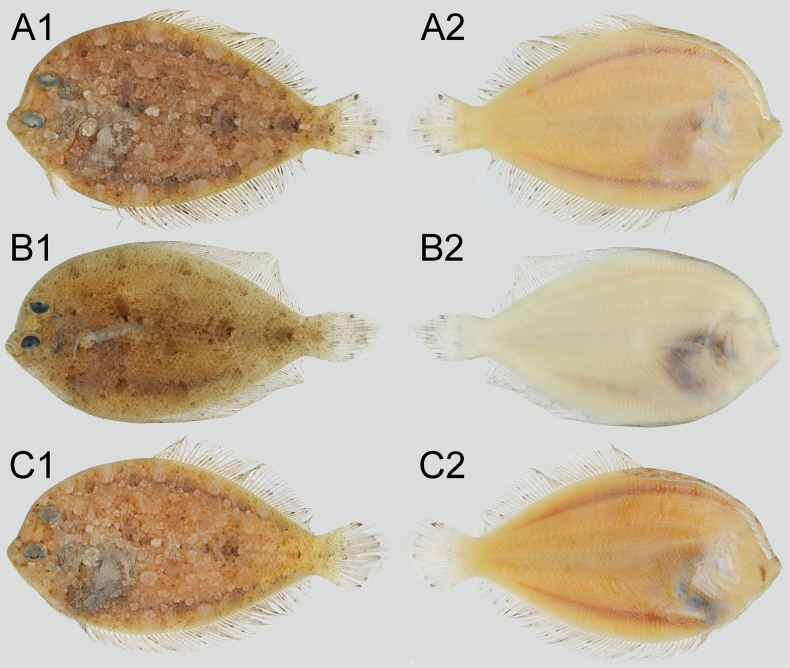
Paratypes of *Crossorhombus pescadores* sp. nov., showing ocular sides (odd numbers) and blind sides (even numbers). **A**. HUMZ 237066, immature male, 74.8 mm SL; **B**. NMMB-P 42263, female, 92.4 mm SL; **C**. NMMB-P 42262, immature female, 58.0 mm SL.

**Figure 3.**
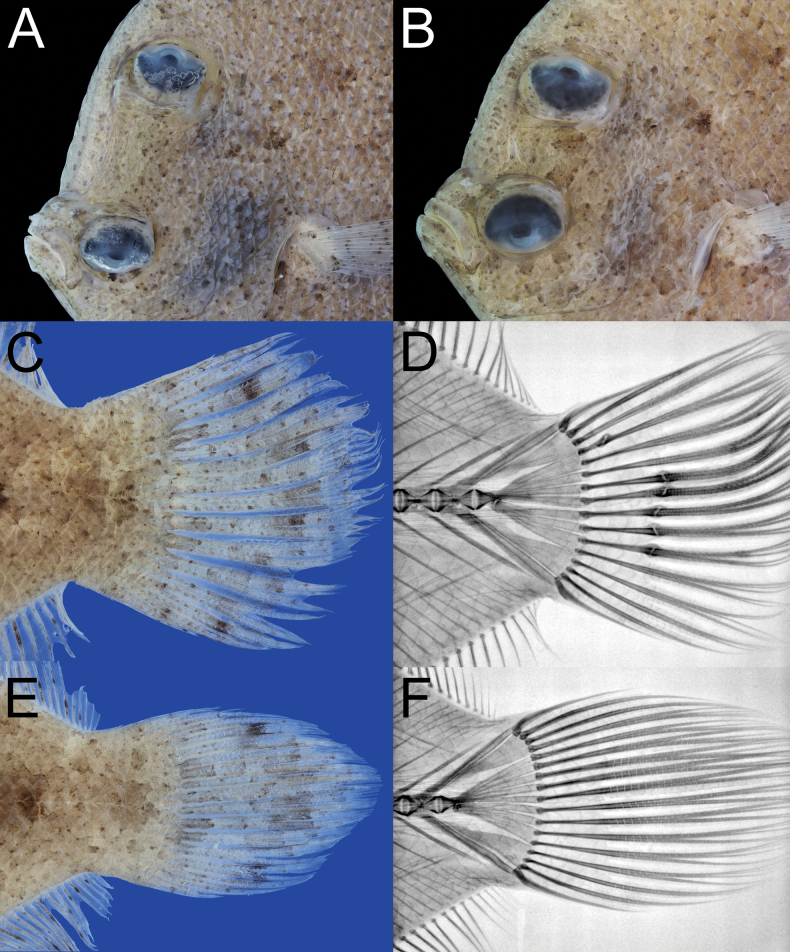
*Crossorhombus pescadores* sp. nov., showing close-up image of head (**A**, **B**), and caudal fin (**C**, **E**), and x-ray image of caudal-fin rays and skeleton (**D**, **F**). **A, C, D**. NMMB-P 42260, holotype, male, 99.8 mm SL; **B**, **E**, **F**. NMMB-P 42263, paratype, female, 92.4 mm SL. Anterior to left. Figures not to scale.

**Figure 4.**
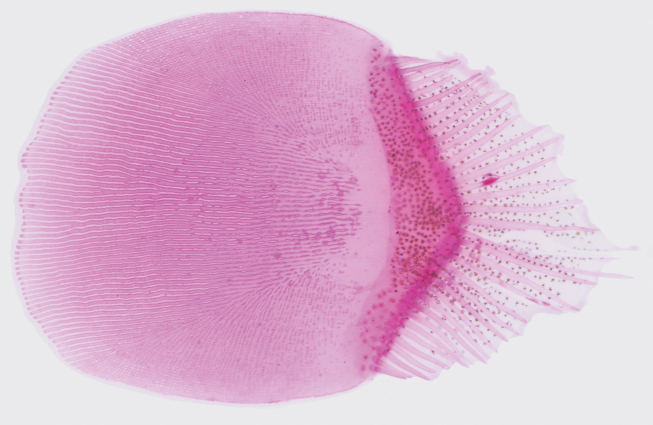
Ocular-side scale (stained with Alizarin Red) of *Crossorhombus pescadores* sp. nov., NMMB-P 42260, holotype, male, 99.8 mm SL. Posterior to right. Figure not to scale.

Figs 1, 2, 3, 4; Table 1

#### New English name:

Pescadores flounder

#### New Chinese name:

澎湖纓鮃

#### Holotype.

• NMMB-P 42260, 99.8 mm SL, male, off Chi-Kan, Baisha, Penghu Islands, Taiwan Strait, Western Pacific Ocean, ca 10–30 m, 9 May 2024.

#### Paratypes.

• NMMB-P 42261, 57.5 mm SL, male; HUMZ 237066, 74.8 mm SL, male; NMMB-P 42262, 58.0 mm SL, female, same as type locality, 14 June 2023; • NMMB-P 42263, 92.4 mm SL, female, collected with holotype.

#### Etymology.

The specific name “pescadores” is the old name of the type locality, meaning “fishermen” in Portuguese. It is treated as a noun in apposition.

#### Diagnosis.

A species of *Crossorhombus* differing from its congeners in having: caudal fin with light-gray band along posterior sub-margin; a pair of small black spots on band at upper and lower fourth rays, respectively; a bluish-black, horizontal triangular marking on blind side of body in male; a series of dark blotches along dorsal and ventral margins of body; combination of some meristic and proportional characters: large head; small upper and lower eyes; slightly longer pectoral-fin on ocular side; fewer dorsal- and anal- fin rays and fewer scales in lateral line and others.

#### Description.

Counts and body proportions in SL and HL are shown in Table 1.

**Table 1.** Comparison among proportional measurements and counts of five species of *Crossorhombus*. Boldfaces show values different (including slight overlaps) from *C. pescadores* sp. nov. Numbers in parentheses are Japanese specimens. M = male, F = female, O = ocular side, B = blind side.

	*C. pescadores* sp. nov.		*C. valderostratus*	*C. howensis*	*C. kobensis*	*C. azureus*
No of specimens	Holotype	Paratypes (*n* = 4)	*n* = 20	*n* =2	*n* = 68	*n* = 20
SL (mm)	99.8 (M)	57.5–74.8 (2M), 58.0–92.4 (2F)	51.3–103.6 (10M, 10F)	62.0–103.0 (1M, 1F)	69.1 (M) (49.2–103.5; 41M, 26F)	80.6–147.2 (10M, 10F)
Proportions, in SL						
Head length	4.06	3.74–3.94	3.65–4.08	**4.19–4.33**	4.09 (3.65–4.28)	**4.15–4.87**
Body depth	1.66	1.66–1.74	1.58–1.85	1.68, 1.68	**1.83 (1.74–2.02)**	**1.75–1.97**
Proportions, in HL						
Snout length	4.47	3.97–5.88	4.32–6.24	4.48–5.06	5.83 (4.91–7.64)	4.88–5.22
Upper-eye diameter	3.32	3.44–3.78	**2.93–3.39**	3.36–3.66	3.13 (2.71–3.50)	3.58–4.13
Lower-eye diameter	3.46	3.37–4.03	**2.93–3.34**	3.15–3.78	3.07 (2.61–3.52)	3.35–4.28
Interorbital width in M	1.88	3.0–3.02	2.36–3.28	2.62	2.32 (2.01–2.65)	2.02–2.55
Interorbital width in F	–	3.69–3.90	**5.28–8.56**	**2.96**	(3.45–12.8)	3.23–4.80
Upper-jaw length (O)	3.32	3.16–3.80	3.04–3.84	3.44–3.72	3.60 (3.01–3.74)	3.71–3.98
Upper-jaw length (B)	4.39	3.65–4.03	3.19–3.92	3.44–3.77	(3.12–3.74)	4.08–4.13
Lower-jaw length (O)	2.46	2.26–2.57	2.25–2.52	2.24–2.43	2.49 (2.13–2.74)	2.49–2.59
Lower-jaw length (B)	2.24	2.21–2.57	2.04–2.31	**2.06–2.18**	(1.81–2.44)	2.37–2.55
Caudal-peduncle depth	1.85	1.89–2.18	1.75–2.15	**1.69–1.87**	2.82 (1.79–2.39)	1.7–1.93
Pectoral-fin length (O) in M	0.68	0.68–1.01	0.61–0.78	0.83	0.78 (0.45–0.98)	**1.25–1.37**
Pectoral-fin length (O) in F	–	1.00–1.02	1.03–1.28	0.75	**(0.71–0.98)**	**1.35–1.45**
Pectoral-fin length (B)	1.55	1.57–2.18	1.56–2.00	1.48–1.61	(1.67–2.67)	1.75–1.81
Pelvic-fin length (O)	1.97	1.97–2.38	1.97–2.55	1.86–1.87	(1.91–2.54)	(1.98–2.60)
Pelvic-fin length (B)	1.85	2.13–2.38	2.06–2.69	1.57–1.97	(2.06–2.61)	(2.13–2.73)
Length of pelvic-fin base (O)	2.18	2.22–2.64	2.01–2.64	1.97–2.48	(2.01–2.69)	(2.13–2.73)
Length of pelvic-fin base (B)	4.24	4.25–6.55	5.38–6.65	5.48–5.53	(3.97–8.18)	(4.07–6.54)
Longest dorsal-fin ray	2.05	1.70–2.07	1.77–1.98	1.85–1.89	(1.60–2.29)	(1.71–2.06)
Longest anal-fin ray	2.02	1.88–2.07	1.59–1.97	1.83–1.90	(1.62–2.27)	(1.70–2.08)
Counts:						
Dorsal-fin rays	82	82–86	82–88	**87–88**	85 (79–86)	**86–92**
Anal-fin rays	62	63–67	62–68	**67–69**	64 (59–67)	**66–72**
Caudal-fin rays (unbranched + branched + unbranched)	2 + 11 + 3 = 16	3 + 11 + 3 = 17	**2 + 13 + 2 = 17**	**2 + 13 + 2 = 17**	3 + 11 + 3 = 17	**2 + 13 + 2 = 17**
Pectoral-fin rays (O)	12	11–12	11–12	11–12	11 (9–11)	12–13
Pectoral-fin rays (B)	9	9	9–10	9	9 (9–10)	9–12
Scales in lateral line	51	44–51	46–55	**53–54**	56 (50–55)	**55–63**
Gill rakers	0 + 6	0 + 6	0–1 + 6–7	0 + 6	0 + 6 (0 + 5–7)	0–4 + 6–8
Vertebrae (abdominal + caudal)	10 + 25	10 + 24–25	10 + 23–25	10 + 25	10 + 25 (10 + 24–26)	10 + 25–26

Data of percent of SL for the holotype are presented, followed by those for the paratypes in parentheses. Head length 24.6 (25.4–26.7); body depth 60.1 (57.4–60.2); snout length 5.5 (4.5–6.6); upper-eye diameter 7.4 (6.9–7.8); lower-eye diameter 7.1 (6.5–7.9); interorbital width 13.1 (8.4–8.9 in males, 6.7–7.2 in females); upper-jaw length 7.4 (6.7–8.4) on ocular side, 5.6 (6.5–7.2) on blind side; lower-jaw length 10.0 (9.9–11.6) on ocular side, 11.0 (9.9–12.1) on blind side; depth of caudal peduncle 13.3 (12.2–14.1); pectoral-fin length 36.1 (26.3–37.4 in males, 25.6–26.7 in females) on ocular side, 15.9 (12.2–16.7) on blind side; pelvic-fin length 12.5 (11.2–13.3) on ocular side, 13.3 (10.7–12.5) on blind side; length of pelvic-fin base 11.3 (9.6–11.8) on ocular side, 5.8 (3.9–6.2) on blind side; length of longest dorsal-fin ray 12.0 (12.3–15.4); length of longest anal-fin ray 12.2 (12.3–15.4); length of middle ray of caudal-fin 20.9 (19.3–24.7).

Body deep and compressed, deepest at a little anterior of middle body, and deeper than half of SL; depth of caudal peduncle 4.5 (4.3–4.7 in paratypes) in body depth. Anterior profile of head steep after deeply concaved before upper margin of lower eye. Head small, a little shorter (longer in paratypes) than 1/4 of SL. Snout protruded, remarkably shorter than eyes. Short spine at tip of snout (spine present in male and absent in female) (Figs 2: A1–C1, 3A, B). Eyes small, anterior margin (middle in female) of upper eye over posterior margin of lower eye, both separated by wide and concaved space (Figs 2: A1–C1, 3A, B); interorbital space 1.88 in HL (3.0–3.02 in males, 3.69–3.9 in females).

Mouth small, upper jaw about equal to eye diameter, posterior end of maxillary extending to anterior margin of lower eye. Teeth on upper jaw in 2 rows, outer row stronger and more spaced than inner ones; those on lower jaw 1 row, about similar to inner teeth on upper jaw; teeth on ocular side stronger and longer than those on blind side. Gill rakers short, blunt, and smooth on posterior margin; no gill rakers on upper limb. Scales on ocular side comb-like ctenoid with a series of long spines along posterior margin (Fig. 4), cycloid on blind side. Lateral line on ocular side strongly curved above pectoral fin, lateral line on blind side absent.

Dorsal fin originating at blind side of snout, anal fin below posterior margin of head, both fins longest at near middle of body. Pectoral fin on ocular side filamentous on upper rays, longest ray longer than head, about 1.5 times of HL (1.0–1.5 in males, 1.0 in females), that on blind side small and short, about half of head. Origin of pelvic fin on ocular side at tip of isthmus, and below middle of lower eye, that on blind side opposite to fifth ray on ocular side. Posterior margin of caudal fin round, upper 2 and lower 3 rays simple (3 and 3), others branched (Fig. 3C–F). Anus opens on blind side above origin of anal fin, genital papilla on opposite side of body.

Caudal skeleton composed of expanded last neural and haemal spines and 4 plates (1^st^ to 4^th^ from upper) of hypurals between these spines; outer 2 plates with grooves from distal to basal, and inner 2 plates clearly divided at distal half (Fig. 3D, F); last neural spine with 1 simple ray, 1^st^ plate with 1 simple ray (2 in all paratypes) and 1 branched ray (1), 2^nd^ plate with 5 branched rays (5), 3^rd^ plate with 4 branched rays (4), 4^th^ plate with 1 branched rays (1) and 2 simple ray (2), and last haemal spine with 1 simple ray.

#### Coloration.

When preserved, body on ocular side dark brownish with a series of dark round blotches along dorsal and ventral sub-margins of body (Figs 1A, 2: A1–C1); an obscure dark blotch at origin, middle, and near base of caudal fin on straight portion of lateral line; dorsal and anal fins pale, scattered with dark specks on each ray; caudal fin with indistinct pale dark band and spots along posterior sub-margin, and with a small distinctly black spot on respective 4^th^ ray from upper and lower (Figs 1, 2: A1–C1, 3C, E). Body on blind side with horizontal triangular bluish-black marking, and anterior portion of marking with wings extending to bases of dorsal and anal fins, and remaining portion not to bases of their fins and caudal fin; anterior central part of the marking extending forward to reach above base of pectoral fin on blind side (Fig. 1B). A thin and small vertical bluish black stripe extending upward from near the rear tip of each jaw (uniformly yellowish white without any bluish-black marking and with or without a faint stripe in young male and female; Figs 1B, 2: A2–C2).

##### Key to species of *Crossorhombus*

**Table:** 

1	Caudal fin with 2 clearly dark bands at near base and distal margin; pectoral fin on ocular side much shorter than HL (1.3–1.4 in HL in males, 1.4–1.5 in females); elliptical bluish-black marking on blind side of body in males	***C. azureus***
–	Caudal fin without 2 clearly dark bands; pectoral fin on ocular side equal to or longer than HL in male (0.45–1.01); heart-, triangular-, and V-shaped bluish-black marking on posterior 2/3 of blind side of body in males	**2**
2	Uppermost and lowermost 3 caudal rays unbranched; body on blind side with horizontal heart-like or triangular bluish-black marking in males	**3**
–	Uppermost and lowermost 2 caudal rays unbranched; body on blind side with horizontal V-shaped bluish-black marking in males	**4**
3	Body narrow, its depth 1.74–2.02 in HL; caudal fin uniformly dark, without any bands and spots; blind side of body having horizontal, heart-shaped, bluish-black marking, and its edge very narrowly spaced from bases of dorsal, anal, and caudal fins in males, but lacking marking in females	***C. kobensis***
–	Body deep, its depth 1.66–1.74 in HL; caudal fin with 1 pair of black spots; blind side of body having horizontal, triangular, bluish-black marking with anterior wings, and wings attached to dorsal- and anal- fin bases but remaining edge narrowly spaced from dorsal, anal, and caudal fins in males, but lacking marking in females	***C. pescadores* sp. nov**.
4	Head large, its length 3.65–4.08 in SL; interorbital width narrow in female, about 5.28–8.56 in HL; pectoral fin in males elongate, longer than HL (0.61–0.78 in HL), in females shorter than HL (1.03–1.28 in HL)	***C. valderostratus***
–	Head small, its length 4.19–4.33 in SL; interorbital width wide in female, about 2.96 in HL; pectoral fin in males and females elongate, longer than HL (0.83 in HL, male; 0.75, female)	***C. howensis***

## Discussion

*Crossorhombus pescadores* sp. nov. can be easily distinguished from the four congeners by having a pair of small black spots on the caudal fin, compared to two black bands in *C. azureus*, and uniformly pale or many dark spots in three other species (Amaoka 2016; Amaoka and Ho 2019). The lower black spot of holotype and large female is smaller than the upper one and less distinct (Figs 1, 2B, 3E), but it is relatively clear in the immature male (Fig. 2A). Therefore, the black spots on caudal fin may fade as it grows.

It also differs from other species in having a horizontal triangular bluish-black marking with anterior wings on the blind side of body in male, compared to an elliptical marking in *C. azureus*, a horizontal V-shape marking in *C. howensis* and *C. valderostratus*, and a heart-shaped marking in *C. kobensis*) (Amaoka and Ho 2019). As shown in Table 1, moreover, the new species differs from *C. howensis* and *C. azureus* in having a larger head, relatively few dorsal- and anal-fin rays and lateral line scales, and 3 simple rays from upper and lower on caudal fin respectively. It also differs from *C. valderostratus* in having smaller eyes and wider interorbital width in females, and from *C. kobensis* in having a deeper body and shorter pectoral-fin length on the ocular side in female.

The dark-blue marking is consistently present in males of all species of *Crossorhombus* and serves as a diagnostic character of the genus. One immature male of *C. pescadores* (74.8 mm SL) has no such marking (Fig. 2: A2). We have observed its appearance in males of *C. kobensis* at approximately 50 mm SL and at approximately 60 mm SL in males of *C. valderostratus* (Amaoka pers. obs.). Consequently, the late appearance of this marking in *C. pescadores* may be also a diagnostic character for it.

It is notable that the holotype has 16 caudal-fin rays (2 simples + 11 branched + 3 simples), but all four paratypes have 17 rays (3 simples + 12 branched + 3 simples). The radiograph of caudal skeleton and rays in the holotype showed damaged condition of five middle rays and the 1^st^ plate abnormally divided into 3 branches which supporting only 2 rays, while all paratypes have 3 divisions supporting 3 rays. Thus, the normal condition of caudal-fin rays is 17 accordingly (Table 1).

### Comparative specimens

***Crossorhombus azureus***. NMMB-P3632, 1 male, 147.2 mm SL, Taiwan, Aug. 1, 1957; NMMB-P8825, 1 male, 4 females, 86.1–136.7 mm SL, Penghu, 32 m, Aug. 30, 2005; NMMB-P22232, 1 male, 2 females 80.6–106.7 mm SL, Ke-tzu-liao, Mar. 11, 2015; NMMB-P22299, 2 females, 94.7–127.2 mm SL, Ke-tzu-liao, Mar. 28, 2015; NMMB-P23183, 2 males, 1 female, 88.0–121.7 mm SL, Ke-tzu-liao, Oct. 6, 2015; NMMB-P23239, 1 male, 90.2 mm SL, Ke-tzu-liao, Feb. 4, 2016; NMMB-P24270, 1 male, 2 females, 88.8–94.0 mm SL, Ke-tzu-liao, Jul. 13, 2016; NMMB-P24367, 1 male, 100.6 mm SL, Ke-tzu-liao, Jun. 27 2016.

***Crossorhombus howensis***. USNM 260394, 1 male, 1 female, 60–103 mm SL, paratypes, Hengchun, Pingtung, 0–6 m, Apr. 23, 1968.

***Crossorhombus kobensis***. NMMB-P22249, 1 male, 69.1 mm SL, Taiwan (possibly Dong Gang, no other data); FAKU 15947, 1 female, 79.8 mm SL, FAKU 15973, 15980, 2 males, 67.1–99.0 mm, Mimase, Kochi Pref., Japan, Feb. 20, 1951; FAKU 25611, 1 male, 97.5 mm SL, Yahatahama, Ehime Pref., Japan, Mar. 13 1956; FAKU 29821, 1 male, 103.5 mm SL, Choshi, Chiba Pref. Japan, Jul. 20, 1958; FAKU 29829–29830, 2 males, 79.3–81.2 mm SL, FAKU 29834, 29837–29838, 29849, 29851, 5 males, 50.5–74.0 mm SL, Mimase, Kochi Pref. Japan, May 23, 1959; FAKU 29866, FAKU 29876, 29878, 29879, 29891–29892, 29894, 7 females, 49.2–70.8 mm SL, Mimase, Kochi Pref. Japan, May 23, 1959.

***Crossorhombus valderostratus***. NMMB-P 22286, 5 males, 4 females, 51.3–70.7 mm, Mar. 28, 2015; NMMB-P 25675, 6 females, 65.2–73.3 mm SL, NMMB-P 25712, 3 males, 60.1–70.5 mm, Jun. 27, 2016, all from Ke-tzu-liao, Kaohsiung, Taiwan.

## Supplementary Material

XML Treatment for Crossorhombus pescadores

## Additional information

### Use of AI

No use of AI was reported.

### Author ORCIDs

Kunio Amaoka https://orcid.org/0009-0005-9439-6994

Yo Su https://orcid.org/0000-0002-3576-9229

Hsuan-Ching Ho https://orcid.org/0000-0003-1154-601X

### Data availability

All of the data that support the findings of this study are available in the main text.
